# Effects of common disinfectants on biofilm formation and eradication in *Klebsiella pneumoniae* with different resistance phenotypes

**DOI:** 10.3389/fcimb.2026.1873119

**Published:** 2026-07-10

**Authors:** Chenlei Huang, Xiaoning Li, Zhenghai Yang, Chenhao Xia, Xintong Zhao, Jinlong Yuan

**Affiliations:** 1 Department of Laboratory Medicine, The First Affiliated Hospital of Wannan Medical University (Yijishan Hospital of Wannan Medical University), Wuhu, Anhui, China; 2 Department of Neurosurgery, The First Affiliated Hospital of Wannan Medical University (Yijishan Hospital of Wannan Medical University), Wuhu, Anhui, China

**Keywords:** biofilm, biofilm eradication, biofilm inhibition, disinfectants, *Klebsiella pneumoniae*

## Abstract

**Objective:**

Biofilm formation is a critical virulence factor that facilitates the persistence and transmission of *Klebsiella pneumoniae* (KP) in healthcare settings, complicating infection control efforts. This study aimed to characterize the biofilm-forming capacity of KP isolates with different resistance phenotypes and to evaluate the antibacterial, biofilm-inhibitory, and biofilm-eradicating activities of commonly used disinfectants.

**Methods:**

Forty-five clinical KP isolates were enrolled, comprising 15 susceptible isolates, 15 extended-spectrum *β-lactamase* (ESBL)-producing isolates, and 15 carbapenem-resistant *Klebsiella pneumoniae* (CRKP) isolates. Minimum inhibitory concentrations (MICs) and minimum bactericidal concentrations (MBCs) of povidone-iodine, chlorhexidine, glutaraldehyde, benzalkonium bromide, and sodium hypochlorite were determined by broth microdilution. Biofilm formation was quantified over 7 days using crystal violet staining (OD_590_). The inhibitory and eradicating effects of chlorhexidine, benzalkonium bromide, and sodium hypochlorite on biofilms were further assessed by crystal violet staining and confocal laser scanning microscopy (CLSM).

**Results:**

MIC and MBC values of the five disinfectants were identical across the three KP groups. Chlorhexidine exhibited the most potent antibacterial activity (MIC: 16 µg/mL; MBC: 32 µg/mL). Biofilm biomass increased progressively in all groups, peaking on day 5. In the inhibition assay, OD_590_ values in disinfectant-treated groups remained consistently lower than those in the positive control group, with 16 µg/mL chlorhexidine, 32 µg/mL benzalkonium bromide, and 1000 µg/mL sodium hypochlorite all significantly reducing biofilm biomass by day 5 (P < 0.05). In the eradication assay, 2000 µg/mL chlorhexidine and 5000 µg/mL sodium hypochlorite significantly reduced OD_590_ values compared with the untreated control (P < 0.05), whereas 2000 µg/mL sodium hypochlorite did not.

**Conclusion:**

Chlorhexidine, benzalkonium bromide, and sodium hypochlorite effectively inhibited biofilm formation in KP isolates regardless of resistance phenotype. Chlorhexidine and benzalkonium bromide exhibited stronger inhibitory activity than sodium hypochlorite. Chlorhexidine and sodium hypochlorite also demonstrated biofilm-eradicating activity, with chlorhexidine showing the greatest overall efficacy.

## 1 Introduction

*Klebsiella pneumoniae* (KP) is a major etiological agent of both healthcare-associated and community-acquired infections, accounting for approximately 10% of nosocomial infections worldwide (Wang et al., 2020). This opportunistic pathogen colonizes the respiratory and gastrointestinal tracts of humans and animals and is capable of causing severe infections, including pneumonia, meningitis, liver abscess, urinary tract infections, and sepsis (Wang et al., 2020; Holt et al., 2015). The widespread and often indiscriminate use of antimicrobial agents has driven the emergence and dissemination of multidrug-resistant KP strains, substantially limiting therapeutic options (Holt et al., 2015).

A key determinant contributing to antimicrobial treatment failure is the ability of KP to form biofilms. Biofilms are structured communities of bacterial cells embedded within a self-produced extracellular polymeric matrix composed primarily of polysaccharides, extracellular DNA, and proteins (Chew et al., 2017). It is estimated that over 90% of bacteria in natural environments exist within biofilms. In hospital environments, biofilms readily colonize both biotic surfaces (e.g., mucosal tissues, wounds) and abiotic surfaces (e.g., catheters, ventilators, sinks), enhancing bacterial survival, promoting resistance to disinfection, and facilitating nosocomial transmission (Zhang et al., 2014; Mbelle et al., 2020; Cubero et al., 2016; Kishibe et al., 2016).

Current research on antibiofilm strategies has largely focused on novel antibiotics, metal-based nanoparticles, and natural bioactive compounds. For instance, antimicrobial coatings on medical devices have been shown to prevent initial bacterial colonization (Russo and Marr, 2019), though concerns remain regarding the potential development of resistance. Silver and other metal-based formulations exhibit activity against both planktonic and biofilm-associated bacteria, yet their clinical application is often constrained by cytotoxicity (Piperaki et al., 2017; Ribeiro et al., 2016). Natural plant-derived compounds may suppress biofilm development by interfering with matrix production, but their antimicrobial potency and stability are often suboptimal (de Oliveira Júnior and Franco, 2020; Gupta et al., 2016). In contrast, the specific efficacy of commonly employed clinical disinfectants against KP biofilms remains incompletely characterized. Therefore, this study was designed to evaluate the antimicrobial activity of five widely used disinfectants and to further investigate the inhibitory and eradicating effects of selected agents on KP biofilms across different resistance phenotypes.

## 2 Materials and methods

### 2.1 Bacterial isolates and reagents

A total of 45 KP strains were isolated from clinical specimens obtained from inpatients in different departments of Yijishan Hospital, Wannan Medical College, between September 2019 and August 2020. The collection included 15 susceptible strains (wild type), 15 ESBL-producing strains, and 15 carbapenem-resistant *Klebsiella pneumoniae* (CRKP) strains. All isolates were identified using the VITEK 2 Compact 60 system. To exclude duplicate strains from the same patient, only one KP isolate per individual was retained during the 12-month collection period. Multiple isolates recovered from a single patient at different time points were discarded, ensuring all 45 strains originated from distinct patients without repetition. KP ATCC 700603 was used as the reference strain, and *Escherichia coli* ATCC 25922 served as the quality-control strain. The study protocol for specimen collection was approved by the hospital Ethics Committee. The five disinfectants, namely povidone-iodine, chlorhexidine, glutaraldehyde, benzalkonium bromide and sodium hypochlorite, were purchased from Shandong Anjie Gaoke Disinfection Technology Co., Ltd. Beef extract broth and Mueller-Hinton (MH) broth were obtained from OXOID, UK.

### 2.2 Preparation of bacterial suspensions

Frozen stock cultures were thawed and subcultured onto chocolate agar plates (which was uniformly used for all isolates to ensure optimal and consistent revival from various clinical specimen sources, although not specific for K. pneumoniae), followed by overnight incubation at 37 °C. Isolated colonies were suspended in 3 mL of sterile physiological saline to achieve a turbidity equivalent to a 0.5 McFarland standard, corresponding to approximately 1.0 × 10_8_ colony-forming units (CFU)/mL.

### 2.3 Determination of minimum inhibitory concentration and minimum bactericidal concentration

MIC and MBC values were determined using the broth microdilution method in 96-well microtiter plates. Stock solutions of each disinfectant were prepared and serially diluted two-fold in MH broth to achieve the desired concentration ranges. Bacterial suspensions adjusted to 0.5 McFarland were further diluted 1:100 in sterile saline. One hundred microliters of the diluted bacterial suspension were added to each well containing 100 µL of the respective disinfectant dilution. Plates were incubated at 37 °C for 18–24 h. Bacterial growth was assessed visually for turbidity and confirmed by measuring optical density at 590 nm (OD_590_) using a microplate reader. The MIC was defined as the lowest concentration of disinfectant that completely inhibited visible bacterial growth. To determine the MBC, a 10 µL aliquot was aspirated from each well showing no visible growth and immediately transferred into an equal volume of the corresponding disinfectant-specific neutralizer to eliminate residual biocidal activity and avoid false-negative colony counts. The neutralization mixtures were incubated at room temperature for 10 minutes with thorough vortexing to ensure complete inactivation. Specifically, a 3% polysorbate-80 (Tween-80) with 0.3% lecithin neutralizer was used for chlorhexidine, benzalkonium bromide, and povidone-iodine; 0.5% glycine neutralizer was applied to quench glutaraldehyde; and 0.5% sodium thiosulfate served as the neutralizer for sodium hypochlorite. After neutralization, a 50 µL aliquot of the neutralized suspension was spread-plated onto Columbia blood agar plates and incubated at 37 °C for 24 h. The effectiveness of each neutralizer was validated in preliminary experiments by comparing the recovery of disinfectant-treated bacteria with untreated controls, confirming that the neutralization procedure was both effective and non-toxic to the test organism. The MBC was defined as the lowest disinfectant concentration that resulted in no visible colonies on the agar surface.

For determination of bactericidal kinetics, bacterial suspensions were exposed to selected concentrations of each disinfectant for varying time intervals (5 min, 10 min, 20 min, 30 min, 1 h, 1.5 h, 2 h, 3 h, 4 h, and 5 h). Following exposure, aliquots were neutralized as appropriate and plated onto Columbia blood agar to assess bacterial survival after overnight incubation at 37 °C.

### 2.4 Biofilm formation assay (crystal violet staining)

The prepared bacterial suspension was diluted 1:200 in fresh beef extract broth to a final concentration of approximately 5 × 10^5^ CFU/mL and aliquoted into sterile 96-well plates or EP tubes (Eppendorf microcentrifuge tubes). Plates were incubated statically at 37 °C to allow biofilm formation. At designated time points, the supernatant was carefully aspirated, and wells were gently washed twice with sterile phosphate-buffered saline (PBS) to remove non-adherent planktonic cells. Adherent biofilms were fixed with methanol for 15 min, stained with 1% (w/v) crystal violet for 20 min, and rinsed thoroughly with sterile distilled water. After air-drying, the retained crystal violet was solubilized with 95% ethanol, and the absorbance was measured at 590 nm using a microplate reader. This procedure was performed daily for 7 consecutive days to construct biofilm growth curves.

### 2.5 Biofilm inhibition assay

To evaluate the inhibitory effect of disinfectants on biofilm development, bacterial suspensions were diluted 1:10, and 50 µL was transferred into sterile EP tubes. Subsequently, 950 µL of beef extract broth containing the designated sub-inhibitory or commonly used concentrations of disinfectants (chlorhexidine: 16 µg/mL; benzalkonium bromide: 32 µg/mL; sodium hypochlorite: 1000 µg/mL) was added. Tubes were incubated statically at 37 °C for 5 days. The medium was carefully replaced every 48 h with fresh broth containing the respective disinfectant to maintain concentration. After 5 days, biofilm biomass was quantified by crystal violet staining as described above. Broth inoculated with bacteria but without disinfectant served as the positive control, while sterile broth served as the blank control. The percentage of biofilm inhibition was calculated as:

Inhibition rate (%) = [(OD_590_ of positive control − OD_590_ of experimental group)/OD_590_ of positive control] × 100%.

### 2.6 Biofilm eradication assay

To assess the eradicating effect on pre-formed mature biofilms, 50 µL of diluted bacterial suspension was added to sterile EP tubes containing 950 µL beef extract broth and incubated at 37 °C for 5 days to allow biofilm maturation. Following incubation, the supernatant was discarded, and the tubes were gently washed twice with sterile PBS to remove planktonic bacteria. Fresh beef extract broth containing the designated concentrations of disinfectants (chlorhexidine: 1000 and 2000 µg/mL; benzalkonium bromide: 1000 µg/mL; sodium hypochlorite: 2000 and 5000 µg/mL) was added to the experimental groups. The control group received an equal volume of drug-free broth. After a further 24 h incubation at 37 °C, the supernatant was carefully discarded, and the tubes were gently washed twice with 200 µL of sterile phosphate-buffered saline (PBS) to remove non-adherent or detached bacteria and matrix debris. The remaining biofilm biomass was then quantified by crystal violet staining as described in Section 2.4.The biofilm eradication rate was calculated as:

Eradication rate (%) = [(OD_590_ of control group − OD_590_ of experimental group)/OD_590_ of control group] × 100%.

### 2.7 Confocal laser scanning microscopy

A small autoclaved coverslip was placed into each EP tube containing bacterial suspension and disinfectant-supplemented broth to serve as a substrate for biofilm growth. After treatment as described in Sections 2.5 and 2.6, the coverslips were gently washed twice with sterile PBS to remove non-adherent cells, mounted onto glass slides, and immediately examined using a Leica SP8 laser scanning confocal microscope. No exogenous fluorescent staining was applied; instead, the intrinsic autofluorescence of K. pneumoniae was captured using excitation at 488 nm and emission between 500 and 550 nm. Images were acquired at 40× magnification and processed with Leica LAS X software (Leica Microsystems, Germany).

For each coverslip, CLSM images were consistently acquired from the bottom layer of the biofilm, defined as the focal plane with maximal autofluorescence intensity immediately adjacent to the coverslip surface. This plane was identified by focusing on the coverslip surface under transmitted light and then fine-tuning to the brightest fluorescent signal (typically 1-2 µm above the surface). The same Z-position was maintained across all treatment groups to ensure comparability.

### 2.8 Statistical analysis

All statistical analyses were performed using SPSS software version 17.0 (SPSS Inc., Chicago, IL, USA). Data are expressed as mean ± standard deviation (SD). Comparisons of biofilm OD_590_ values among multiple groups were performed using one-way analysis of variance (ANOVA) followed by the least significant difference (LSD) *post hoc* test. Time-course data were analyzed using repeated-measures ANOVA. Figures were generated using GraphPad Prism version 6.0 (GraphPad Software, San Diego, CA, USA). A two-sided P value < 0.05 was considered statistically significant.

## 3 Results

### 3.1 Determination of MIC and MBC for five disinfectants

All disinfectants were diluted to the aforementioned concentrations. The MIC and MBC were determined via visual turbidity observation and OD_590_ measurement. The MIC and MBC of each disinfectant remained consistent among the three bacterial groups, and the detailed results are presented in **Table 1**.

**Table 1:** MIC and MBC of different disinfectants.

	Povidone-iodine	Glutaraldehyde	Chlorhexidine	Benzalkonium bromide	Sodium hypochlorite
MIC (µg/ml)	256	2500	16	32	1000
MBC (µg/ml)	512	5000	32	64	2000

MIC, minimum inhibitory concentration; MBC, minimum bactericidal concentration

### 3.2 Determination of bactericidal time for each disinfectant

The MBC and routinely used concentrations of each disinfectant were evaluated against bacterial suspensions at exposure times of 5 min, 10 min, 20 min, 30 min, 1 h, 1.5 h, 2 h, 3 h, 4 h, and 5 h. After exposure, the treated suspensions were inoculated onto Columbia blood agar plates and incubated at 37 °C for 24 h to assess bacterial survival. The minimum exposure time required for complete killing at each concentration was recorded.

Overall, most disinfectants exhibited rapid bactericidal activity against the three KP groups under the tested conditions. Chlorhexidine, benzalkonium bromide, glutaraldehyde, and povidone-iodine all achieved complete killing within 5 min at their tested effective concentrations. Specifically, chlorhexidine at 1000 µg/mL and 2000 µg/mL completely killed the susceptible, ESBL-producing, and CRKP groups within 5 min. Benzalkonium bromide at 500, 1000, and 3000 µg/mL also produced complete killing within 5 min in all three groups. Similarly, glutaraldehyde at 10,000 µg/mL and 20,000 µg/mL and povidone-iodine at 2000 µg/mL and 5000 µg/mL eradicated all three groups within 5 min.

In contrast, sodium hypochlorite showed relatively slower bactericidal activity. At 10,000 µg/mL, complete killing was achieved within 5 min in the susceptible and CRKP groups, whereas 10 min was required for the ESBL-producing group. At 5000 µg/mL, sodium hypochlorite required 10 min to completely kill all three groups. Compared with the other disinfectants tested, sodium hypochlorite required either a higher concentration or a longer exposure time to achieve the same bactericidal effect (**Table 2**).

**Table 2:** Bactericidal time of different disinfectants against bacterial strains.

		Susceptible	ESBLs	CRKP
Chlorhexidine	32 µg/ml 100 µg/ml 500 µg/ml 1000 µg/ml 2000 µg/ml	5 h 2 h 10 min 5 min 5 min	4 h 2 h 10 min 5 min 5 min	2 h 1 h 10 min 5 min 5 min
**Benzalkonium Bromide**	64 µg/ml 100 µg/ml 500 µg/ml 1000 µg/ml 3000 µg/ml	1 h 1 h 5 min 5 min 5 min	2 h 20 min 5 min 5 min 5 min	3 h 20 min 5 min 5 min 5 min
Glutaraldehyde	5000 µg/ml 10000 µg/ml 20000 µg/ml	5 min 5 min 5 min	20 min 5 min 5 min	1 h 5 min 5 min
Povidone-Iodine	512 µg/ml 1000 µg/ml 2000 µg/ml 5000 µg/ml	4 h 1 h 5 min 5 min	4 h 1 h 5 min 5 min	4 h 2 h 5 min 5 min
Sodium Hypochlorite	2000 µg/ml 5000 µg/ml 10000 µg/ml	1 h 10 min 10 min	1 h 10 min 5 min	2 h 10 min 10 min

ESBL, extended-spectrum β-lactamase. CRKP, carbapenem-resistant *K. pneumoniae*

### 3.3 Biofilm detection of *Klebsiella pneumoniae*

**Figure 1:**
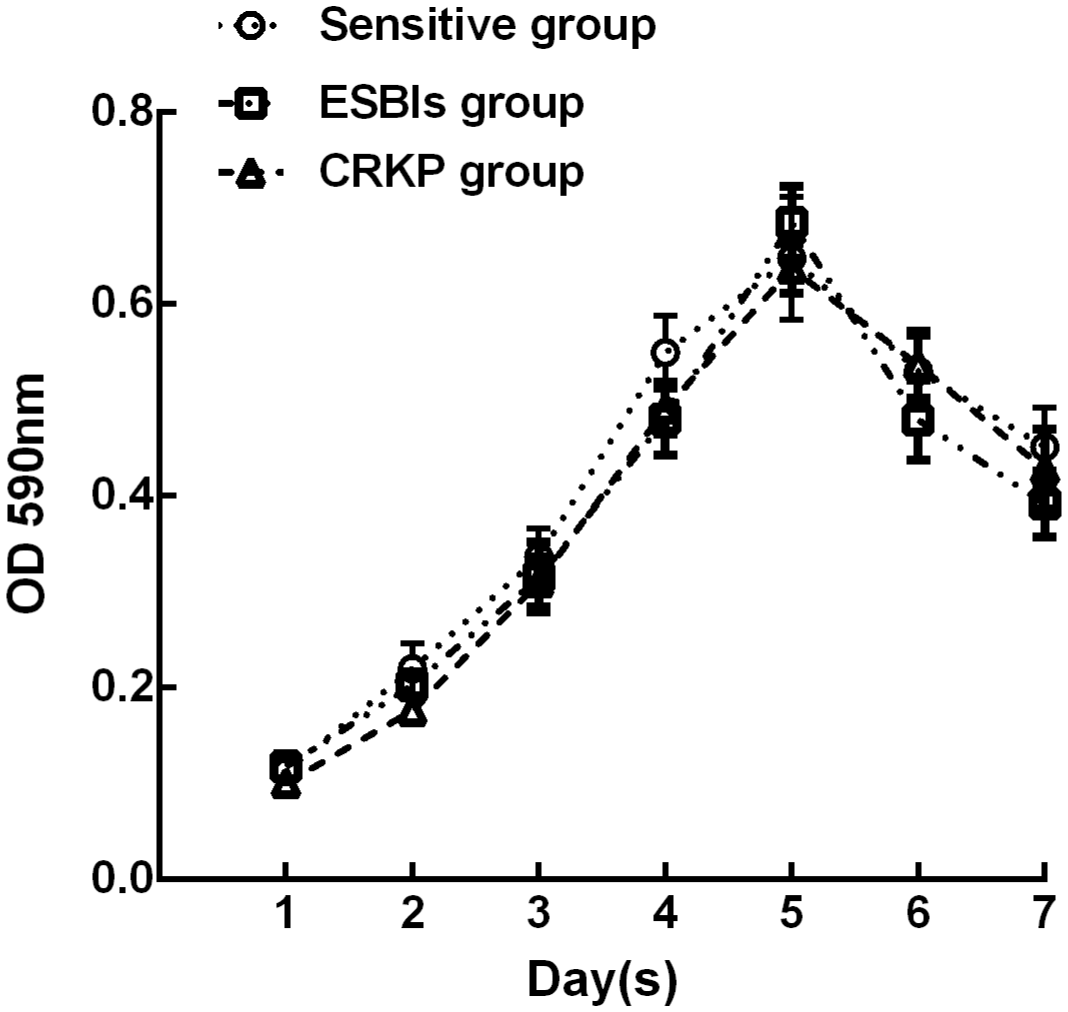
Time course of biofilm formation in the three KP groups. Biofilm formation was detectable on day 1 and gradually increased, reaching maturity on day 5 in all three groups.

Biofilm formation was detectable on day 1 in all three bacterial groups and increased progressively over time, reaching a maximum on day 5 before declining thereafter. OD_590_ values were measured daily from day 1 to day 7. As shown in **Table 3**, no statistically significant difference in day-5 biofilm biomass was observed among the three groups (P > 0.05). The corresponding growth curves are shown in **Figure 1**.

**Table 3:** Biofilm biomass of the three bacterial groups on day 5 (Mean ± SD).

Bacterial Group	Biofilm Biomass on Day 5	F-value	P-value
Susceptible	0.658 ± 0.073	0.213	0.809
ESBLs	0.640 ± 0.081		
CRKP	0.649 ± 0.077		

ESBL, extended-spectrum β-lactamase; CRKP, carbapenem-resistant *K. pneumoniae*.

### 3.4 Biofilm inhibition assay

**Figure 2:**
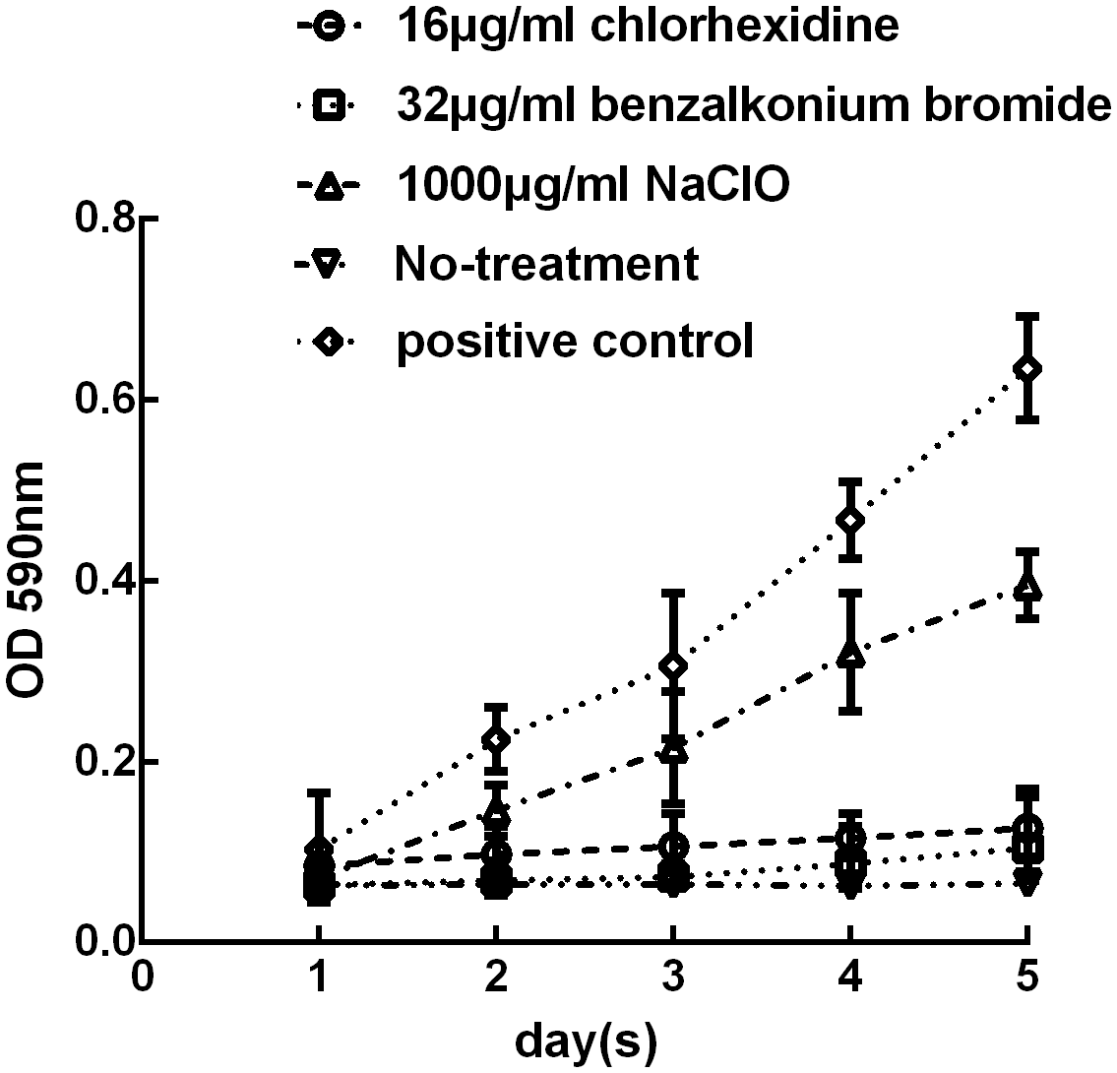
Biofilm growth curve of susceptible KP after treatment with different disinfectants. Biofilm biomass increased over time but remained lower than the positive control.

Based on the potent bactericidal activity observed, chlorhexidine, benzalkonium bromide, and sodium hypochlorite were selected for biofilm inhibition experiments. As shown in **Figure 2** (using the susceptible group as a representative example), biofilm biomass increased progressively over the 5-day culture period; however, the OD_590_ values in the disinfectant-treated groups remained consistently lower than those in the positive control group. Similar trends were also observed for the ESBL-producing and CRKP isolates.

**Figure 3:**
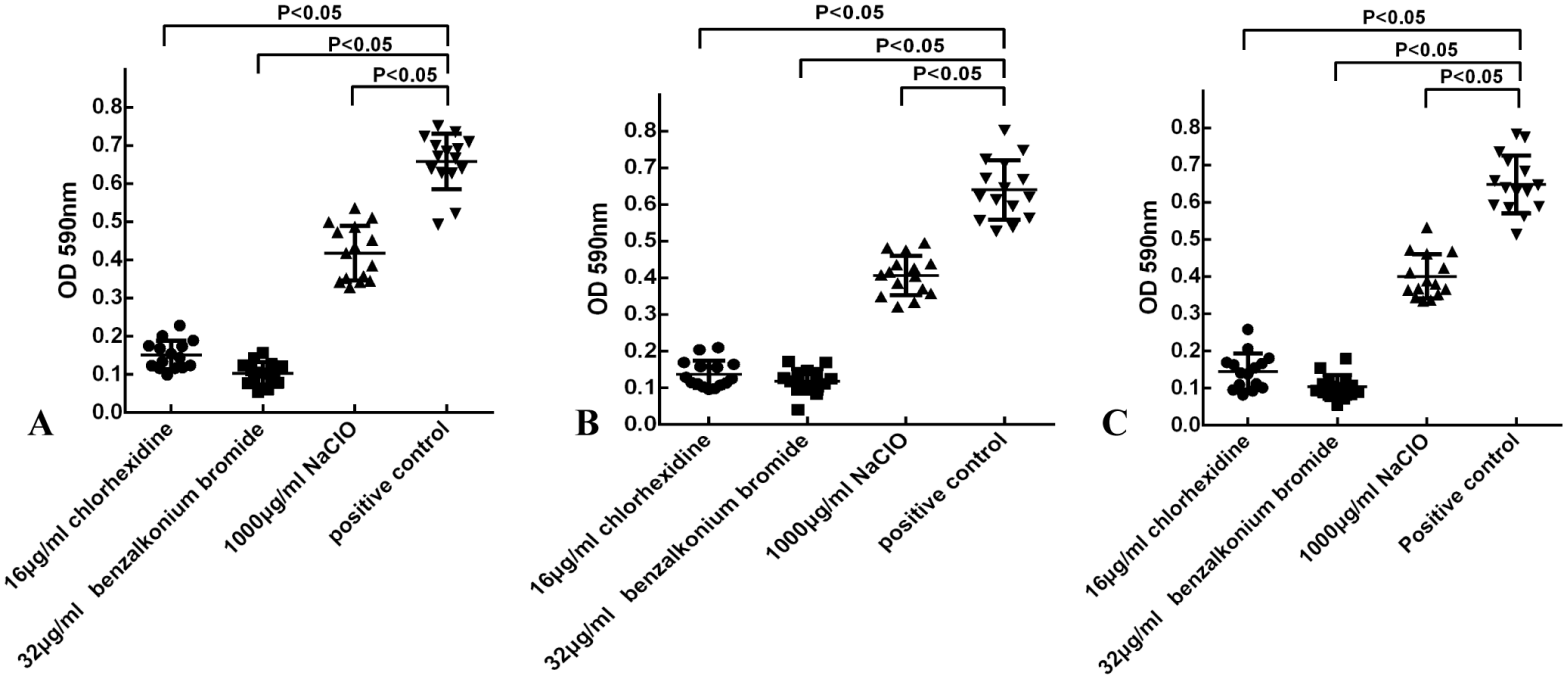
Inhibitory effects of disinfectants on KP biofilms. **(A)** Susceptible group; **(B)** ESBL group; **(C)** CRKP group. Bars represent mean OD_590_ ± SD. P < 0.05 compared to positive control.

**Figure 4:**
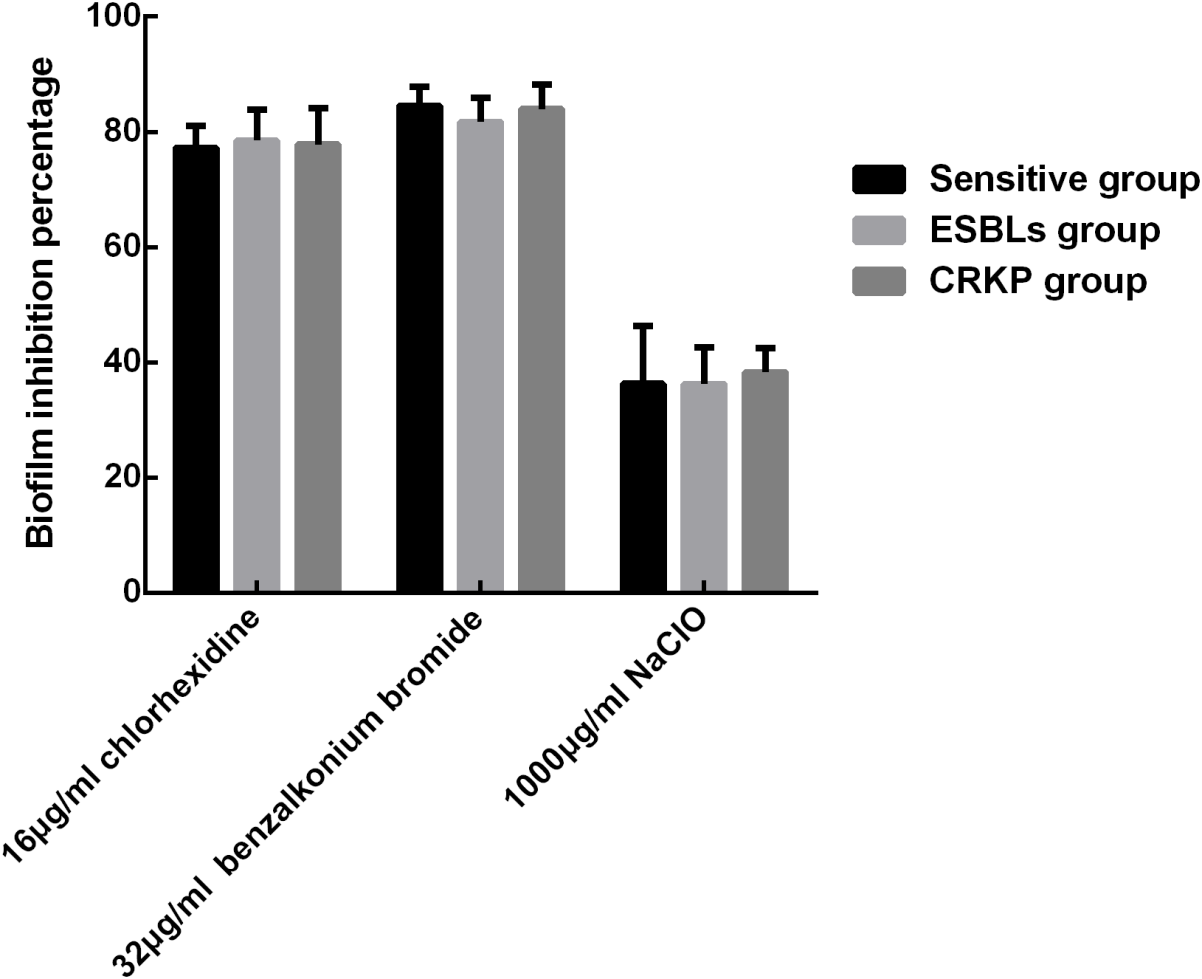
Biofilm inhibition rates following disinfectant treatment. Higher inhibition rates were observed for chlorhexidine and benzalkonium bromide compared to sodium hypochlorite.

On day 5, treatment with 16 µg/mL chlorhexidine, 32 µg/mL benzalkonium bromide, and 1000 µg/mL sodium hypochlorite resulted in significantly reduced biofilm biomass across all three KP groups compared with the untreated positive control (P < 0.05) (**Figure 3**). The corresponding inhibition rates are presented in **Figure 4**. Overall, 32 µg/mL benzalkonium bromide and 16 µg/mL chlorhexidine exhibited markedly stronger inhibitory effects than 1000 µg/mL sodium hypochlorite.

### 3.5 Biofilm eradication assay

Following 24 h of exposure to different concentrations of disinfectants, the residual biomass of 5-day-old mature KP biofilms was quantified by crystal violet staining. In the susceptible group, the mean OD_590_ values after treatment with 1000 µg/mL and 2000 µg/mL chlorhexidine were 0.593 ± 0.065 and 0.519 ± 0.074, respectively, both of which were significantly lower than that of the blank control group (0.658 ± 0.073, P < 0.05). In contrast, treatment with 1000 µg/mL benzalkonium bromide resulted in a mean OD_590_ value of 0.605 ± 0.067, which did not differ significantly from that of the blank control (P > 0.05). Similarly, 2000 µg/mL sodium hypochlorite did not significantly reduce biofilm biomass (0.612 ± 0.067, P > 0.05), whereas 5000 µg/mL sodium hypochlorite significantly reduced the OD_590_ value to 0.561 ± 0.055 (P < 0.05).

**Figure 5:**
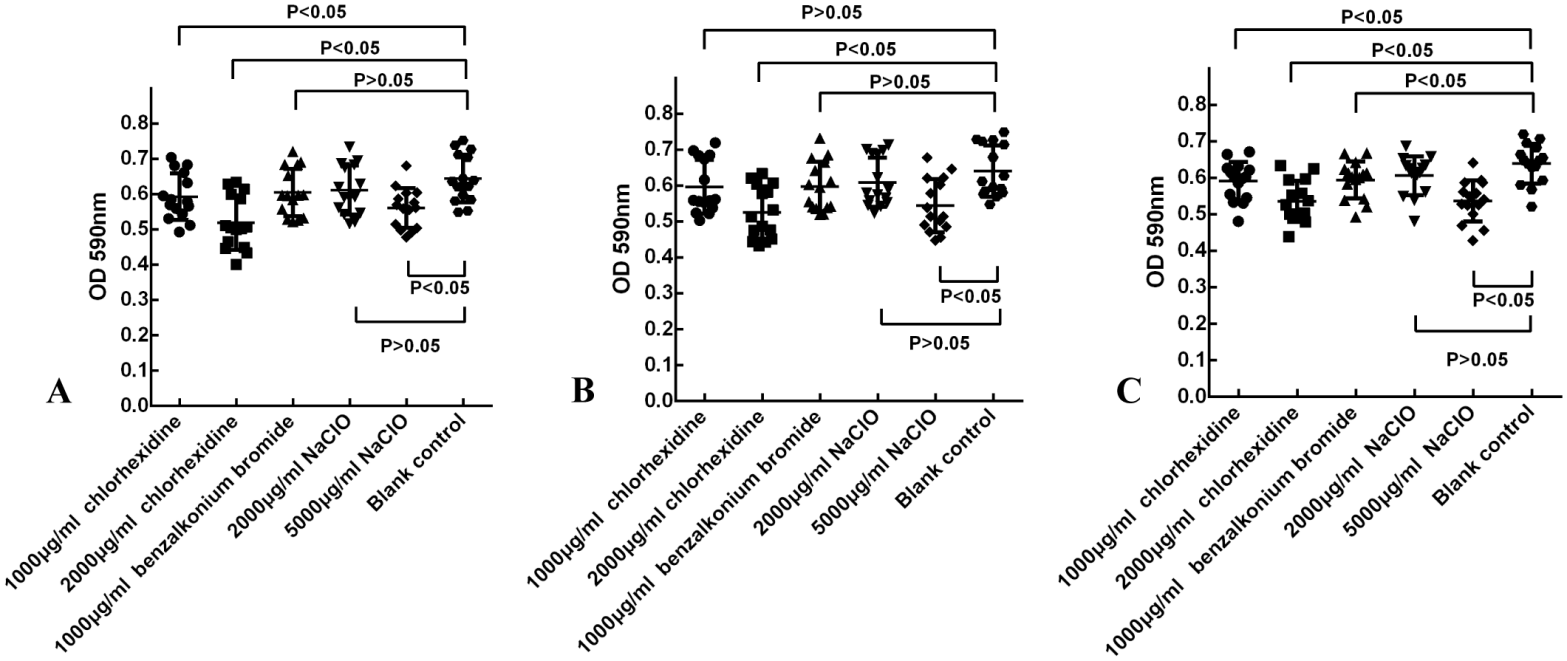
Eradication effects of disinfectants on pre-formed KP biofilms. **(A)** Susceptible group; **(B)** ESBL group; **(C)** CRKP group. Bars represent mean OD590 ± SD. P < 0.05 compared to blank control.

A similar trend was observed in the ESBL-producing and CRKP groups. In the ESBL-producing group, significant reductions in biofilm biomass were observed only after treatment with 2000 µg/mL chlorhexidine and 5000 µg/mL sodium hypochlorite, with OD_590_ values of 0.525 ± 0.073 and 0.579 ± 0.056, respectively, compared with 0.640 ± 0.081 in the blank control group (P < 0.05). By contrast, 1000 µg/mL chlorhexidine, 1000 µg/mL benzalkonium bromide, and 2000 µg/mL sodium hypochlorite did not produce statistically significant reductions in this group. In the CRKP group of *Klebsiella pneumoniae*, compared with the blank control group, the differences observed in the 1000 µg/mL chlorhexidine group (0.591 ± 0.057), 2000 µg/mL chlorhexidine group (0.536 ± 0.056), 1000 µg/mL benzalkonium bromide group (0.594 ± 0.051), and 5000 µg/mL sodium hypochlorite group (0.538 ± 0.059) were statistically significant (P < 0.05). However, the difference in the 2000 µg/mL sodium hypochlorite group was not statistically significant (P > 0.05) (**Figure 5**).

**Figure 6:**
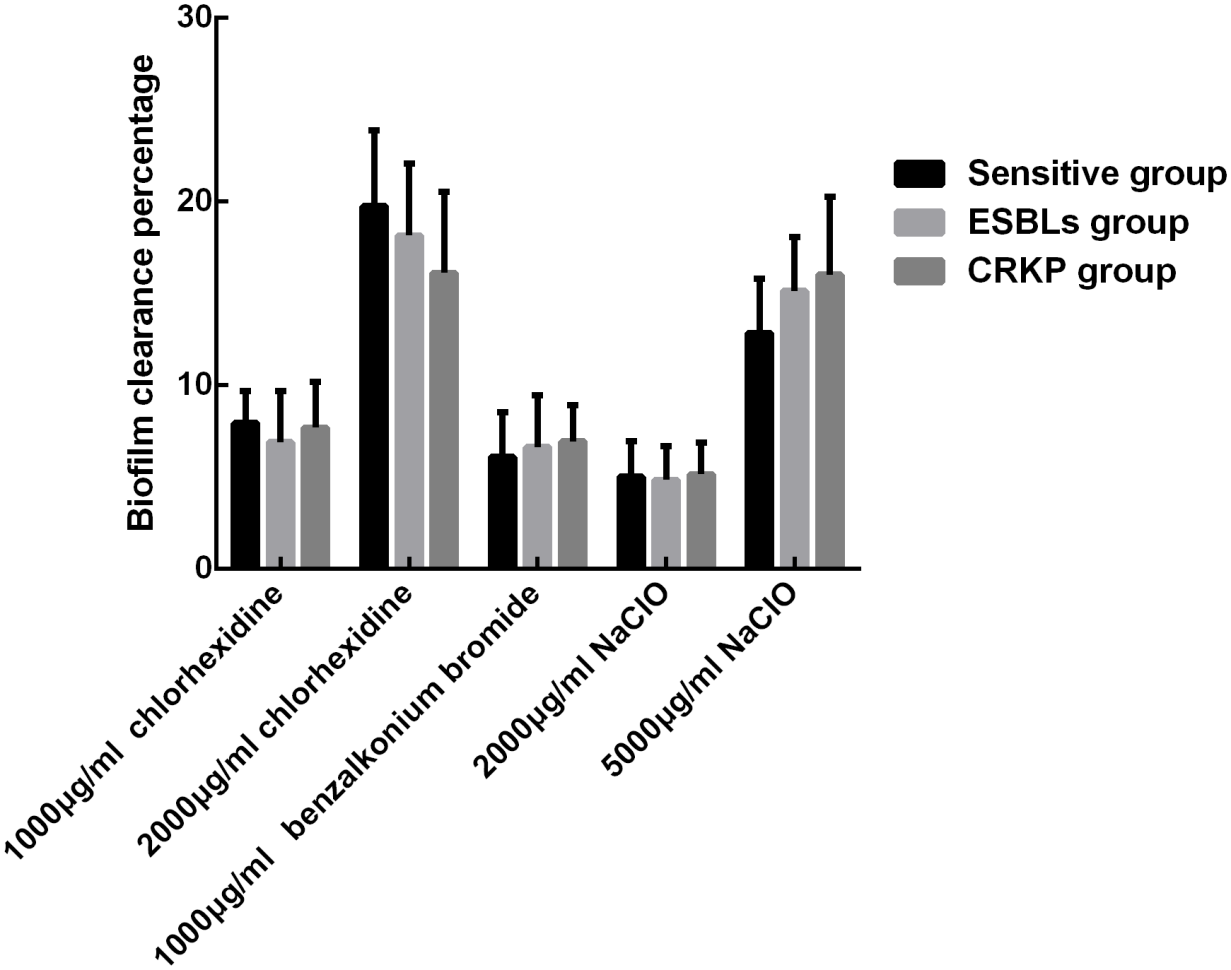
Biofilm eradication rates following 24-h disinfectant exposure. The highest eradication rates were achieved with 2000 µg/mL chlorhexidine and 5000 µg/mL sodium hypochlorite.

Overall, mature KP biofilms showed limited susceptibility to lower disinfectant concentrations. Among the tested conditions, 2000 µg/mL chlorhexidine and 5000 µg/mL sodium hypochlorite consistently produced greater reductions in biofilm biomass across the three resistance phenotypes, whereas 1000 µg/mL benzalkonium bromide showed little effect on established biofilms. The comparative eradication rates are presented in **Figure 6**.

### 3.6 CLSM visualization

**Figure 7:**
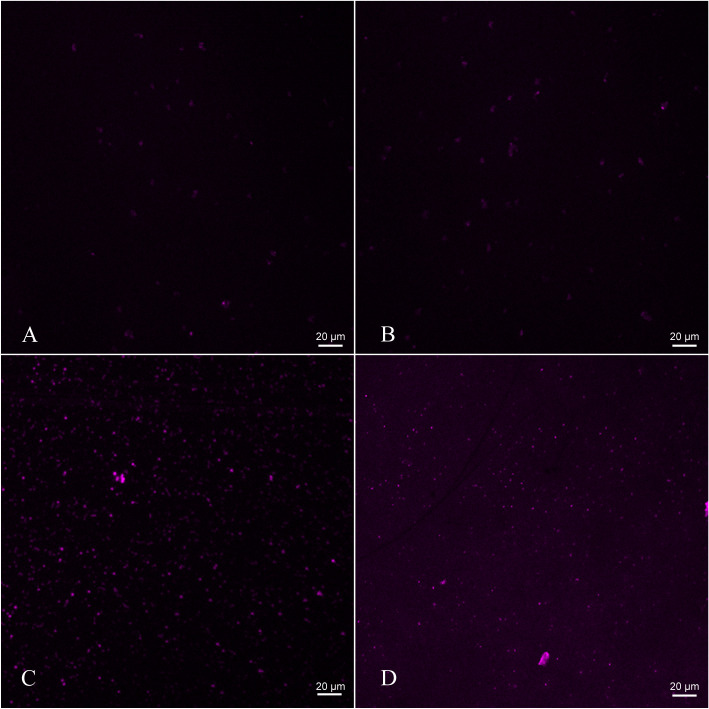
CLSM images of biofilm inhibition in susceptible KP (40×). **(A)** 32 µg/mL benzalkonium bromide; **(B)** 16 µg/mL chlorhexidine; **(C)** 1000 µg/mL sodium hypochlorite; **(D)** Positive control. Scale bar = 20 µm.

CLSM images corroborated the crystal violet staining findings. In the inhibition assay, the positive control displayed a dense, interconnected network of extracellular polysaccharides with aggregated bacterial clusters. Detailed microstructural observation revealed that the positive control formed thick continuous EPS wrapping large bacterial aggregates, while chlorhexidine and benzalkonium bromide induced severe fragmentation of EPS networks and scattered distribution of tiny bacterial clusters; sodium hypochlorite only partially loosened biofilm structure with obvious residual bacterial aggregation. In contrast, treatment with 16 µg/mL chlorhexidine, 32 µg/mL benzalkonium bromide, or 1000 µg/mL sodium hypochlorite resulted in a marked reduction in matrix deposition and a looser biofilm architecture with scattered bacterial distribution (**Figure 7**).

**Figure 8:**
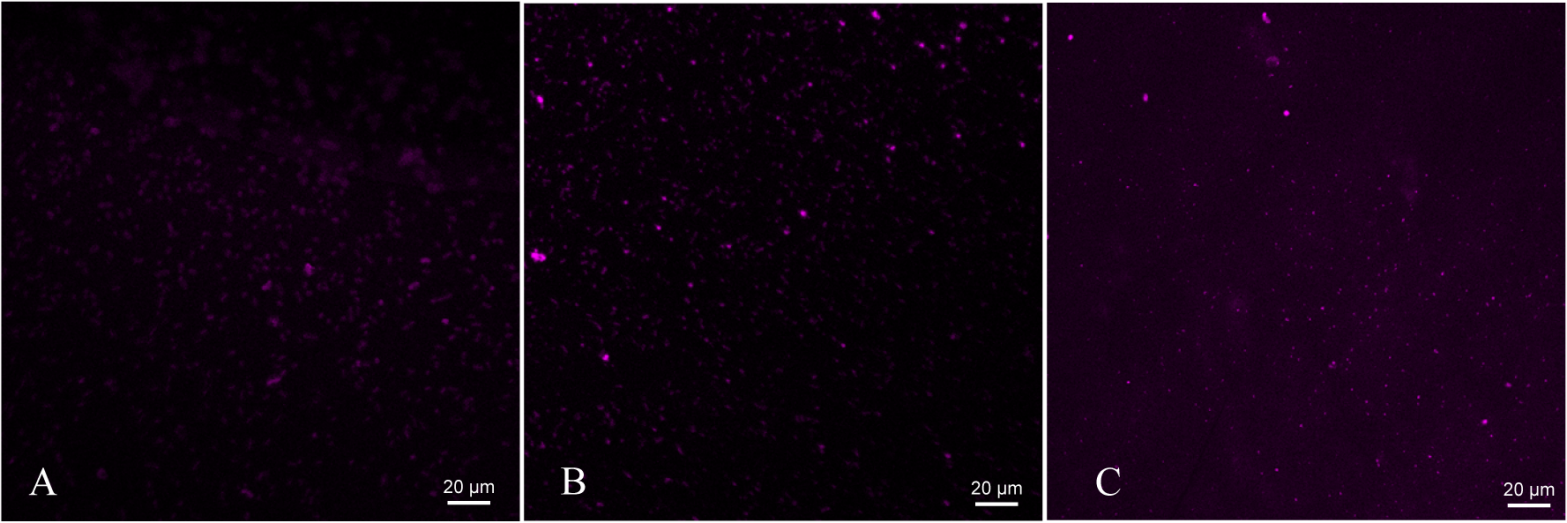
CLSM images of biofilm eradication in susceptible KP (40×). **(A)** 2000 µg/mL chlorhexidine; **(B)** 5000 µg/mL sodium hypochlorite; **(C)** Blank control. Scale bar = 20 µm.

In the eradication assay, the blank control (mature biofilm treated with drug-free broth) exhibited a robust, mesh-like biofilm structure. For mature biofilms, 2000 µg/mL chlorhexidine caused complete collapse of compact layered biofilm and decomposition of large bacterial clusters into individual cells; 5000 µg/mL sodium hypochlorite partially disrupted biofilm architecture but retained minor aggregated microcolonies and residual extracellular matrix. Treatment with 2000 µg/mL chlorhexidine or 5000 µg/mL sodium hypochlorite dramatically reduced the extracellular matrix, leaving only dispersed individual bacterial cells (**Figure 8**).

## 4 Discussion

KP is a major pathogen associated with nosocomial infection and substantial morbidity and mortality. It harbors multiple antimicrobial resistance determinants, including genes encoding extended-spectrum *β-lactamases* and carbapenemases, and readily acquires multidrug resistance, making infections increasingly difficult to treat (Mbelle et al., 2020). Biofilm formation is a critical contributor to this resistant phenotype, as it enables KP to evade host immune clearance and tolerate antimicrobial exposure, thereby promoting persistent infection and treatment failure (Lahiri et al., 2021). In the present study, we compared day-5 biofilm biomass among susceptible, ESBL-producing, and CRKP isolates and further evaluated the inhibitory and eradicating effects of several commonly used disinfectants on KP biofilms. Our results show that these disinfectants differ substantially in their antibiofilm activity against KP isolates with different resistance phenotypes.

Among the disinfectants tested, chlorhexidine and benzalkonium bromide showed the lowest MIC and MBC values, indicating stronger antibacterial activity against *K. pneumoniae*. In the bactericidal-time assay, povidone-iodine at 2000 µg/mL, glutaraldehyde at 10,000 µg/mL, chlorhexidine at 1000 µg/mL, and benzalkonium bromide at 500 µg/mL all achieved complete killing within 5 min in all three bacterial groups. Notably, povidone-iodine and glutaraldehyde require far higher effective concentrations to exert bactericidal effects, and their clinical application scenarios are limited to skin mucosal antisepsis and high-level disinfection of medical devices respectively, which are less relevant to environmental biofilm contamination caused by K. pneumoniae. Therefore, only chlorhexidine, benzalkonium bromide and sodium hypochlorite with broader environmental disinfection applications were selected for subsequent biofilm inhibition and eradication assays, while povidone-iodine and glutaraldehyde were merely detected for MIC and MBC as reference disinfectants. These findings suggest that chlorhexidine and benzalkonium bromide possess relatively high antimicrobial efficiency under the tested conditions.

Chlorhexidine is a well-established antiseptic widely used in clinical practice, particularly in oral care (Papas et al., 2012; Varoni et al., 2012). Its antimicrobial effects are mainly attributed to disruption of the bacterial lipid bilayer, interference with cell adhesion, and inhibition of biofilm formation (Ahmed et al., 2016). At higher concentrations, chlorhexidine can cause severe membrane damage and rapid leakage of intracellular contents (Zhou and Nanayakkara, 2021). Schmidt et al. reported that 0.05% chlorhexidine was effective against Staphylococcus epidermidis biofilms after only 1 min of exposure, whereas sodium hypochlorite, triple-antibiotic solution, and saline were ineffective under the same conditions (Schmidt et al., 2018). Biswas et al. further demonstrated that chlorhexidine exerts antimicrobial effects against carbapenem-resistant Acinetobacter baumannii by inducing reactive oxygen species, triggering lipid peroxidation, and disrupting membrane integrity (Biswas et al., 2019). In addition, chlorhexidine-based combinations have shown promising antibiofilm activity. For example, chlorhexidine-conjugated gold nanoparticles inhibited both biofilm formation and metabolic activity in KP in a time-dependent manner and affected established biofilms (Ahmed et al., 2016), while combinations of chlorhexidine with nanosilver were effective against multispecies biofilms in root-canal models (Tülü et al., 2021). Consistent with these reports, our results indicate that chlorhexidine strongly inhibits biofilm formation in KP strains with different resistance profiles and partially eradicates mature biofilms.

Benzalkonium bromide, a quaternary ammonium disinfectant with cationic surfactant properties, primarily acts by disrupting the cytoplasmic membrane and, in Gram-negative bacteria, the outer membrane, leading to leakage of intracellular components and cell lysis. This effect may be related to electrostatic interactions between the cationic disinfectant and negatively charged components of the biofilm matrix, which could facilitate penetration into the extracellular polymeric substance and interfere with early biofilm stability. However, studies on its effects on bacterial biofilms remain limited. Geraldes et al. demonstrated that benzalkonium chloride at concentrations of 4–16 mg/L inhibited biofilm formation by Acinetobacter baumannii, possibly through modulation of efflux pump activity and reduction of bacterial surface hydrophobicity (Migliaccio et al., 2024). Other studies have shown that benzalkonium chloride can kill Staphylococcus aureus biofilms on polycarbonate surfaces and that this activity may be enhanced by mild positive pressure (Shamaila et al., 2022). Our results are in line with these observations and further suggest that benzalkonium bromide is more effective at preventing KP biofilm formation than at eliminating established biofilms.

Sodium hypochlorite is a chlorine-based disinfectant with broad-spectrum antimicrobial activity. Previous studies have shown that it reduces the viability of Staphylococcus aureus and Pseudomonas aeruginosa biofilms and significantly inhibits biofilm formation at working concentrations (Lineback et al., 2018; Gutierrez et al., 2014; Otter et al., 2015; Tiwari et al., 2018; Yanling et al., 2018). It also exhibits activity against mature biofilms. Plutzer et al. found that 4% sodium hypochlorite was the only tested agent capable of eliminating Enterococcus faecalis biofilms (Plutzer et al., 2018), and Nair et al. observed that static exposure to sodium hypochlorite frequently resulted in complete dissolution of biofilms *in vitro* (Nair et al., 2005). Petridis et al. and Clegg et al. further suggested that biofilm removal depends on the amount of active sodium hypochlorite delivered to a given surface area, either by increasing concentration or optimizing exposure conditions (Petridis et al., 2019; Clegg et al., 2006). A similar trend was observed in the present study. Silva, Gama, and Badaró et al. also reported that higher concentrations and/or longer exposure times improved the eradication of Candida albicans biofilms (Silva et al., 2011; Gama et al., 2015; Badaró et al., 2021). Together, these findings suggest that increasing concentration or prolonging contact time may enhance the eradication of KP biofilms by sodium hypochlorite.

The antibiofilm activity of sodium hypochlorite may involve at least two mechanisms. First, because most natural biofilms develop under near-neutral pH conditions (da Silva et al., 2011), the highly alkaline environment of sodium hypochlorite solutions (pH > 11) may alter microbial metabolism and surface interactions, thereby interfering with adhesion and destabilizing biofilm structure. Second, sodium hypochlorite can denature proteins within the extracellular matrix and inactivate essential bacterial enzymes, ultimately leading to irreversible bacterial death (Lineback et al., 2018). However, more direct mechanistic studies are still needed to clarify how sodium hypochlorite disrupts specific structural components of the biofilm matrix.

To verify that OD_590_ accurately reflected biofilm biomass, confocal laser scanning microscopy was performed on bacteria adhered to coverslips. In the positive control group, biofilms appeared as interconnected networks with aggregated clusters. In contrast, after treatment with chlorhexidine, benzalkonium bromide, or sodium hypochlorite, the biofilm architecture was markedly attenuated, with only scattered colonies remaining. These microscopic observations were consistent with the crystal violet staining results and further support the reliability of the quantitative OD_590_ measurements.

Overall, the present findings indicate that commonly used disinfectants differ significantly in their activity against KP biofilms. Chlorhexidine showed the most consistent performance, with strong effects on both biofilm inhibition and partial eradication, whereas benzalkonium bromide was more effective in preventing biofilm formation than in removing mature biofilms. Sodium hypochlorite also demonstrated antibiofilm activity, particularly at higher concentrations, highlighting the importance of adequate exposure conditions in practical disinfection. These results provide experimental support for the rational selection of disinfectants for the control of KP-related biofilm contamination in clinical settings.

## 5 Limitations

This study has several inherent limitations. First, all disinfectants were evaluated using a unified broth microdilution system containing nutrient media (Mueller-Hinton broth and beef extract broth). While this standardized experimental setup ensured consistent assay conditions and comparability of results across the five disinfectants and three *Klebsiella pneumoniae* resistance phenotypes, the organic constituents in the nutrient broth—including proteins, amino acids, and carbohydrates—readily react with and deplete the free chlorine released by sodium hypochlorite. Such chlorine consumption substantially reduces the effective concentration of sodium hypochlorite during incubation, indicating that the antibacterial, biofilm-inhibitory, and biofilm-eradicating activities determined in this study are conservative and likely underestimated. Consequently, this liquid medium model is suboptimal for characterizing the true disinfection efficacy of sodium hypochlorite, and its applicability for evaluating chlorine-based disinfectants warrants further validation.

Second, the *in vitro* carrier-based biofilm model used in this study cannot fully replicate the complexity of *Klebsiella pneumoniae* biofilms that develop *in vivo* or on medical device surfaces under authentic clinical conditions. Third, although this study systematically compared the antibiofilm performance of different disinfectants, the underlying molecular mechanisms remain unexplored and require further investigation.

Fourth, the crystal violet staining method used to quantify biofilm biomass does not distinguish between viable and non-viable cells within the biofilm matrix. Therefore, the reductions in OD_590_ observed in the inhibition and eradication assays reflect the overall removal or disruption of the biofilm structure rather than a direct measure of bacterial viability. However, given that the disinfectant concentrations used in the eradication assays (particularly 2000 µg/mL chlorhexidine and 5000 µg/mL sodium hypochlorite) were substantially higher than their corresponding MBC values against planktonic bacteria (**Table 1**), it is reasonable to infer that the residual biofilm biomass was largely composed of non-viable cells or matrix debris. Nevertheless, direct viability assessment methods, such as resazurin reduction assay or CFU determination of scraped biofilms, should be incorporated in future studies to complement the current findings.

For future research, we recommend using low-organic or inorganic buffer systems (e.g., sterile phosphate-buffered saline) or standard surface carrier models with minimal organic interference to assess the actual efficacy of sodium hypochlorite more accurately. Additionally, experimental models incorporating simulated organic contaminants are highly desirable to better replicate real-world hospital environmental scenarios.

## 6 Conclusion

In conclusion, chlorhexidine, benzalkonium bromide, and sodium hypochlorite all exhibited inhibitory effects on biofilm formation in KP isolates with different resistance phenotypes. Among them, chlorhexidine and benzalkonium bromide showed stronger antibiofilm inhibitory activity than sodium hypochlorite, whereas chlorhexidine demonstrated the most consistent overall performance by exerting both strong biofilm inhibition and partial eradication effects. These findings provide experimental evidence for the rational selection of disinfectants for controlling KP biofilm-associated contamination in clinical settings.

## Data Availability

The original contributions presented in the study are included in the article/supplementary material. Further inquiries can be directed to the corresponding author.
